# Identification and validation of fatty acid metabolism-related lncRNA signatures as a novel prognostic model for clear cell renal cell carcinoma

**DOI:** 10.1038/s41598-023-34027-9

**Published:** 2023-04-29

**Authors:** Cheng Shen, Zhan Chen, Jie Jiang, Yong Zhang, Xinfeng Chen, Wei Xu, Rui Peng, Wenjing Zuo, Qian Jiang, Yihui Fan, Xingxing Fang, Bing Zheng

**Affiliations:** 1 Department of Urology, The Second Affiliated Hospital of Nantong University, Nantong, 226001 China; 2 Medical Research Center, The Second Affiliated Hospital of Nantong University, Nantong, China; 3 Department of Orthopedics, The Second Affiliated Hospital of Nantong University, Nantong, China; 4 Department of Paediatric, Chinese Medicine Hospital of Rudong, Nantong, China; 5 Department of Pathogenic Biology, School of Medicine, Nantong University, Nantong, China; 6 Department of Nephrology, The Second Affiliated Hospital of Nantong University, Nantong, 226001 China

**Keywords:** Cancer, Computational biology and bioinformatics, Immunology, Nephrology, Oncology

## Abstract

Clear cell renal cell carcinoma (ccRCC) is a main subtype of renal cancer, and advanced ccRCC frequently has poor prognosis. Many studies have found that lipid metabolism influences tumor development and treatment. This study was to examine the prognostic and functional significance of genes associated with lipid metabolism in individuals with ccRCC. Using the database TCGA, differentially expressed genes (DEGs) associated with fatty acid metabolism (FAM) were identified. Prognostic risk score models for genes related to FAM were created using univariate and least absolute shrinkage and selection operator (LASSO) Cox regression analyses. Our findings demonstrate that the prognosis of patients with ccRCC correlate highly with the profiles of FAM-related lncRNAs (AC009166.1, LINC00605, LINC01615, HOXA-AS2, AC103706.1, AC009686.2, AL590094.1, AC093278.2). The prognostic signature can serve as an independent predictive predictor for patients with ccRCC. The predictive signature's diagnostic effectiveness was superior to individual clinicopathological factors. Between the low- and high-risk groups, immunity research revealed a startling difference in terms of cells, function, and checkpoint scores. Chemotherapeutic medications such lapatinib, AZD8055, and WIKI4 had better outcomes for patients in the high-risk group. Overall, the predictive signature can help with clinical selection of immunotherapeutic regimens and chemotherapeutic drugs, improving prognosis prediction for ccRCC patients.

## Introduction

Renal cell carcinoma (RCC) is one of the most prevalent kinds of urinary tract cancer, impacting approximately 430,000 individuals worldwide in 2020^1^. Approximately 70% of all RCC cases are clear cell renal cell carcinoma (ccRCC), the most common and malignant subtype^2^. Overall, the prognosis is uncertain due to tumor recurrence or metastasis during the course of the disease, with roughly one-third of patients presenting with metastases at the time of diagnosis^3^. Metastatic ccRCC is always associated with a high mortality rate^4,5^. Patients with RCC that metastasizes have a dismal 12% OS rate at 5 years^6–8^. Despite improvements in diagnosis, screening, surgery, and pharmaceutical treatment, the clinical outcome of RCC remains poor. Hence, there is an urgent need to create more accurate prognostic models given the significant morbidity and mortality of ccRCC.

Reprogramming of energy metabolism is a characteristic of cancer development and stimulates cell growth and proliferation^9^. Increasing data suggest that fatty acid metabolism plays a critical role in metabolic reprogramming, influencing cell membrane formation, energy storage, and synthesis of signaling molecules^10,11^. According to previous research, the rate-controlling enzyme ACLY, which is involved in the first stage of lipid production, can promote colon cancer spread^12^. Increased lipolysis and fatty acid production in cervical cancer patients lead to lymphatic dissemination via activation of the nuclear factor kB (NF-kB) signaling pathway^9,13^. Acute myeloid leukemia cells are more likely to survive when their fatty acid oxidation is activated^14^, and changes in fatty acid metabolism may affect the immune response and the effectiveness of chemotherapy and radiotherapy^15,16^. Previous research has demonstrated that fatty acids can influence the phenotype and function of invading immune cells and may result in immunosuppression^17^. Recent research has demonstrated glycolytic reprogramming in RCC causes down-regulated METTL14 to accumulate BPTF and increase super-enhancers and distal lung metastasis^18^. Furthermore, RCC reprogrammes glutamine, tryptophan, and arginine metabolism to support tumor growth and carcinogenesis^19^. However, the prognostic and potential therapeutic implications of genes implicated in fatty acid metabolism in ccRCC patients, particularly in immunotherapy, are the subject of surprisingly few investigations.

Long noncoding RNAs (lncRNAs) play a crucial role in metabolic reprogramming in controlling metabolism-related pathways^20–22^. For instance, lncRNA-GLCC1 was discovered to reprogramme glycolytic metabolism and was connected to colorectal cancer prognosis^23^. In lung adenocarcinomas, ccat1/FABP5 facilitates tumor growth by regulating fatty acid metabolism^24^. LINC01606 is an oncogene to encourage tumor cell stemness while defending colon cancer cells against iron-induced cell death^25^. For several cancer types, CDKN2B-AS1 is seen as a prospective biomarker or therapeutic target^26^.

As a result, we conducted this extensive, systematic study to identify genes involved in fatty acid metabolism using a weighted gene coexpression network analysis (WGCNA) approach and then created a lncRNA risk profile containing the eight FAM-related lncRNAs (AC009166.1, LINC00605, LINC01615, HOXA-AS2, AC103706.1, AC009686.2, AL590094.1, AC093278.2). We tested their value in predicting prognosis and response to chemotherapy and immunotherapy in patients with ccRCC. The findings of this study add to our understanding of how FAM affects ccRCC and will help ccRCC patients to receive more effective individualized care.

## Results

### Analysis of FAM-related gene enrichment

**Figure 1:**
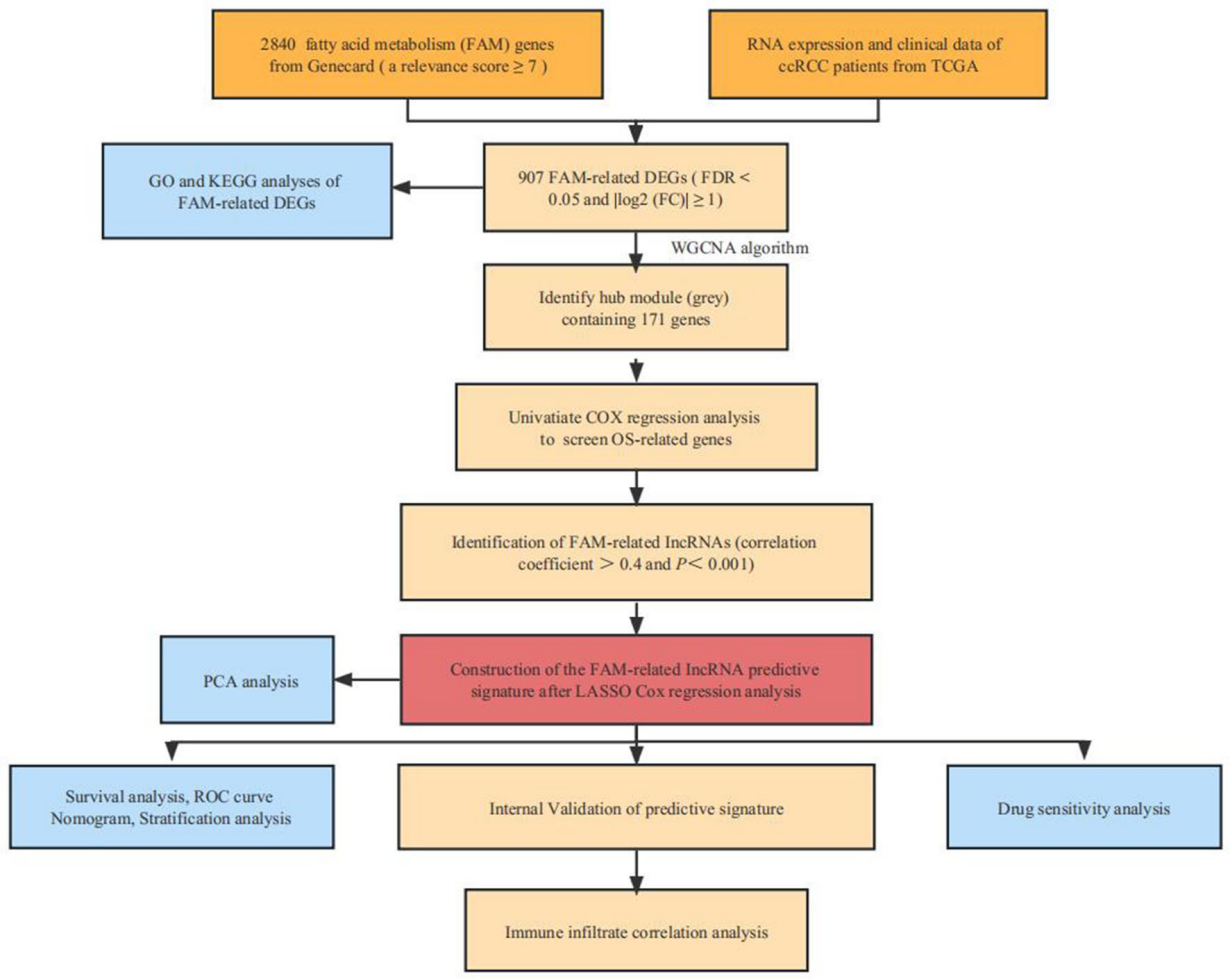
Flow chart of the study. ccRCC, renal clear cell carcinoma; TCGA, The Cancer Genome Atlas; DEGs, differentially expressed genes; GO, Gene Ontology; KEGG, Kyoto Encyclopedia of Genes and Genomes; lncRNAs, long noncoding RNAs; ROC, receiver operating characteristic.

**Figure 2:**
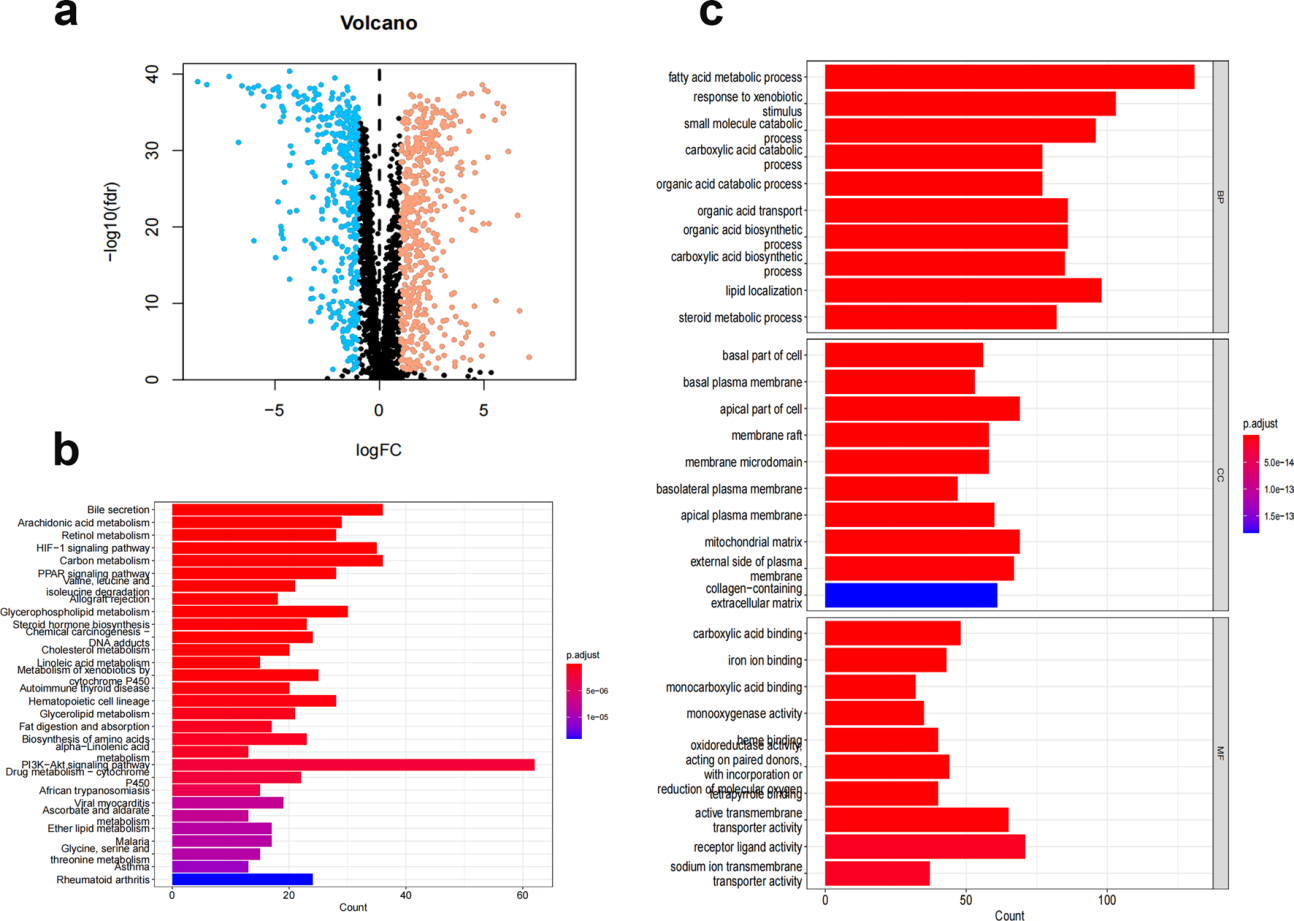
GO and KEGG analysis of FAM-associated DEGs in cancerous and adjacent tissues. (**a**) The 907 FAM-related genes in ccRCC. Yellow dots indicate upregulated genes, and blue dots indicate downregulated genes. (**b**) KEGG analysis of FAM-associated DEGs. (**c**) GO analysis of FAM-associated DEGs. GO, Gene Ontology; KEGG, Kyoto Encyclopedia of Genes and Genomes; DEGs, differentially expressed genes; FC, fold change; FDR: false discovery rate; BP, biological process; CC, cellular component; MF, molecular function.

By following the steps illustrated in Fig. 1, we screened a total of 907 FAM-associated DEGs, 421 of which were upregulated and 486 downregulated (Fig. 2a and Supplementary File 1). Then, we determined the pathways in which the FAM-associated DEGs are involved using GO and KEGG enrichment analyses, including the PI3K-Akt signaling pathway, the HIF-1 signaling system, bile secretion, and the carbon metabolism-related signaling pathway in cancer. KEGG analysis is mainly the Metabolic Signaling pathway (Fig. 2b). Analysis of DEG enrichment in biological processes found that these genes play a significant role in FAM, response to environmental stimuli, and lipid localization. In the cellular component category, the DGEs are primarily abundant in the mitochondrial matrix near the apex of the cell. The DEGs are primarily enriched in receptor‒ligand activity, active transmembrane transporter activity, and carboxylic acid binding GO activities within the molecular function category (Fig. 2c). Most of the genes upregulated affect lipid metabolism.

### Web module mining WGCNA

**Figure 3:**
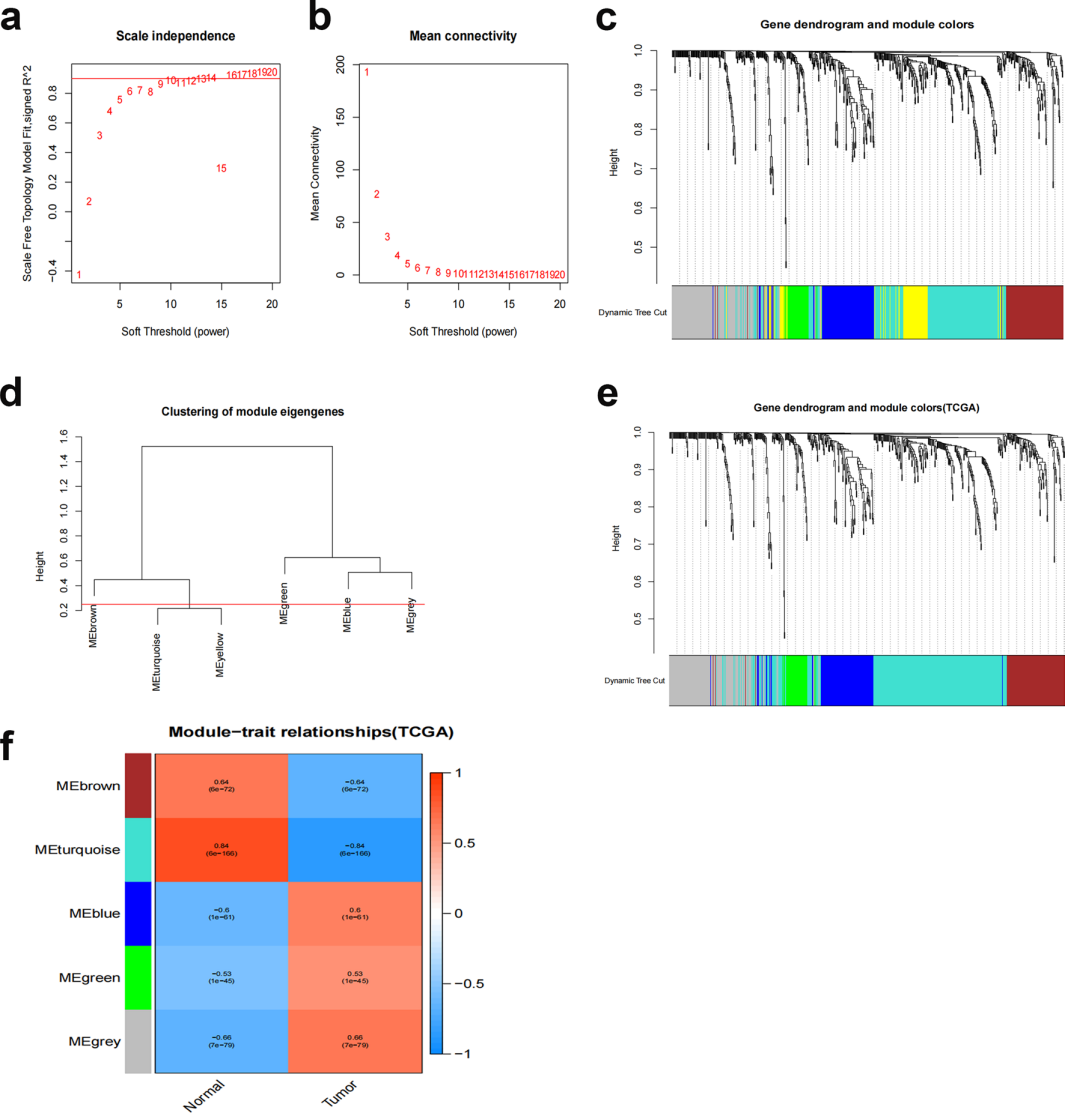
WGCNA network module mining. (**a**,**b**) Determination of the optimal soft threshold by network topology analysis. The scale-free topology threshold of 0.9 is satisfied when β = 9. (**c**) Gene dendrogram and nodal color of WGCNA. (**d**) Hierarchical clustering analysis of the WGCNA module. (**e**) Gene dendrograms are based on clustering. (**f**) Correlation between modules.

WGCNA was applied to the DEGs filtered from TCGA. The connection between genes in the gene network satisfied the scale-free network distribution when the soft threshold power β = 9 (scale-free R2 = 0.9) was initially estimated using the scale-free topology criterion (Fig. 3a,b). Then, a phylogenetic tree was constructed to mine coexpression modules (cut height ≥ 0.25) (Fig. 3c). Using hierarchical clustering, modules were analyzed, and modules on the same branch displayed similar gene expression patterns (Fig. 3d). Four coexpression modules were created by combining the related gene modules (Fig. 3e). Among them, the tumor score and the gray module showed a strong positive correlation (cor = 0.53; P = 9e−14) (Fig. 3f). Consequently, 171 important genes from the gray modules were chosen for additional study (Supplementary File 2).

### Differentially expressed lncRNAs associated with FAM genes used to develop predictive characteristics

**Figure 4:**
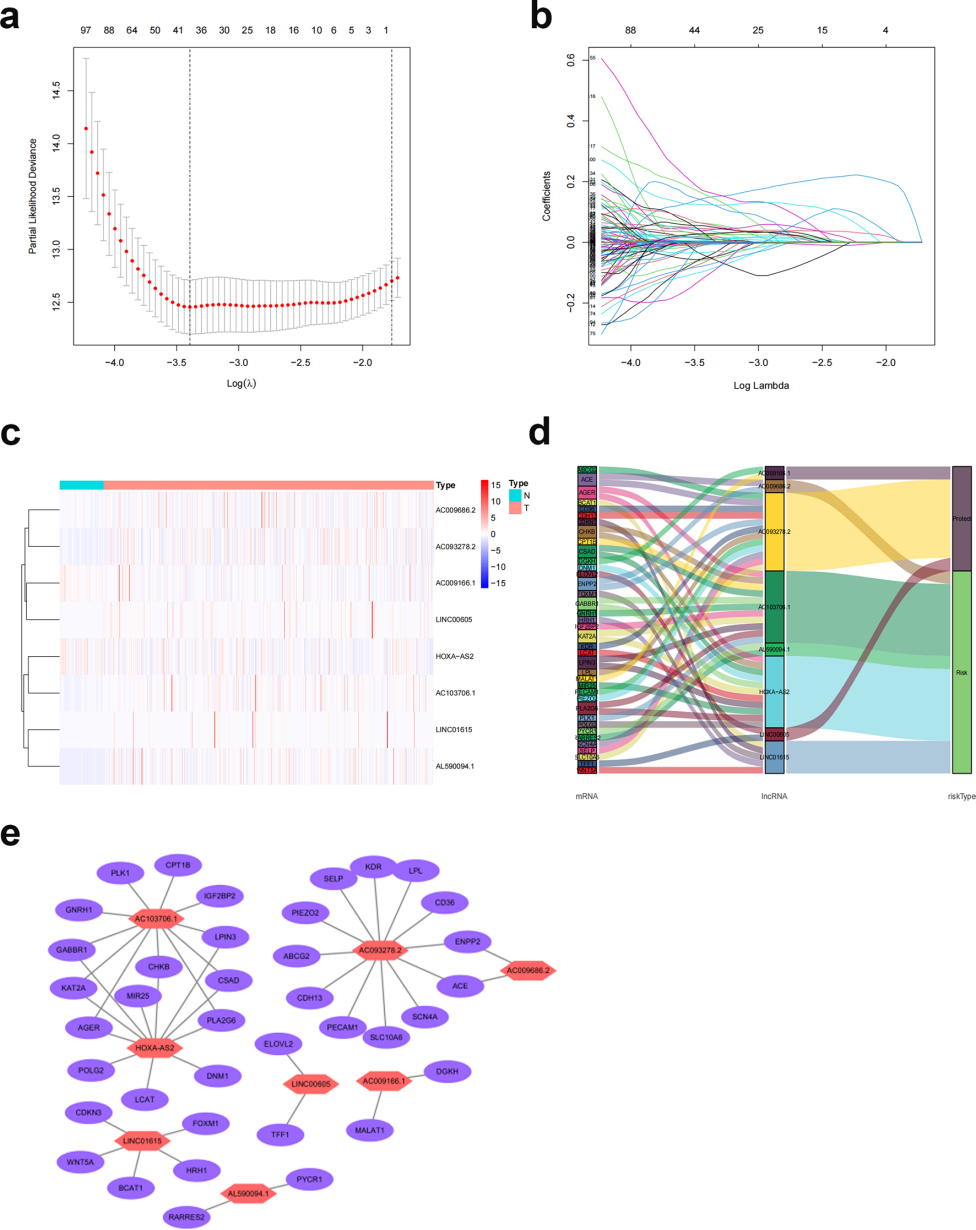
Analysis of the expected signal, including screening, expression levels, and lncRNA‒mRNA networks for the eight lncRNAs related to FAM. (**a**) Tenfold cross-validation error rate plot. (**b**) LASSO coefficient profiles of FAM-associated lncRNAs. (**c**) Heatmap (red represents high expression, whereas blue represents low expression) of the eight FAM-associated lncRNAs between the normal (brilliant blue) and the tumor tissues (red) (R software (version 4.2.1, URL: http://www.r-project.org)). (**d**) Prognostic FAM-associated lncRNA coexpression network. (**e**) A Sankey diagram of lncRNAs linked with prognostic FAM. Long non-coding RNAs, lncRNAs; renal clear cell carcinoma, ccRCC.

We examined 1258 lncRNAs linked to differentially expressed FAM genes. There were 555 lncRNAs (Supplementary File 3) related to prognosis in ccRCC patients, according to single-variable Cox regression analysis. We created a predictive model utilizing LASSO Cox regression and 1000-fold cross-validation to predict the prognosis of patients with ccRCC (Fig. 4a,b). Eight lncRNAs (AC009166.1, LINC00605, LINC01615, HOXA-AS2, AC103706.1, AC009686.2, AL590094.1, AC093278.2) related to FAM were used to create predictive characteristics. The expression levels of the eight FAM-related lncRNAs in ccRCC patients are displayed in Fig. 4c (Supplementary File 4). We used the R packages ggalluvial and Cytoscape to further visualize lncRNAs. Thirty-eight lncRNA‒mRNA pairs were used for the coexpression network (Fig. 4d). LINC01615, HOXA-AS2, AC103706.1, AC009686.2, and AL590094.1 were found to be risk factors and AC009166.1, AC093278.2, and LINC00605 protective factors (Fig. 4e). The risk score was calculated as follows: Risk score = (− 0.53 × AC009166.1 expression) + (− 0.64 × LINC00605 expression) + (0.35 × LINC01615 expression) + (0.53 × HOXA-AS2 expression) + (0.55 × AC103706.1 expression) + (0.71 × AC009686.2 expression) + (0.71 × AC009686.2 expression) + (0.41 × AL590094.1 expression) + (− 0.36 × AC093278.2 expression).

### Analysis of the relationship between prognosis and prognostic traits of individuals with ccRC

**Figure 5:**
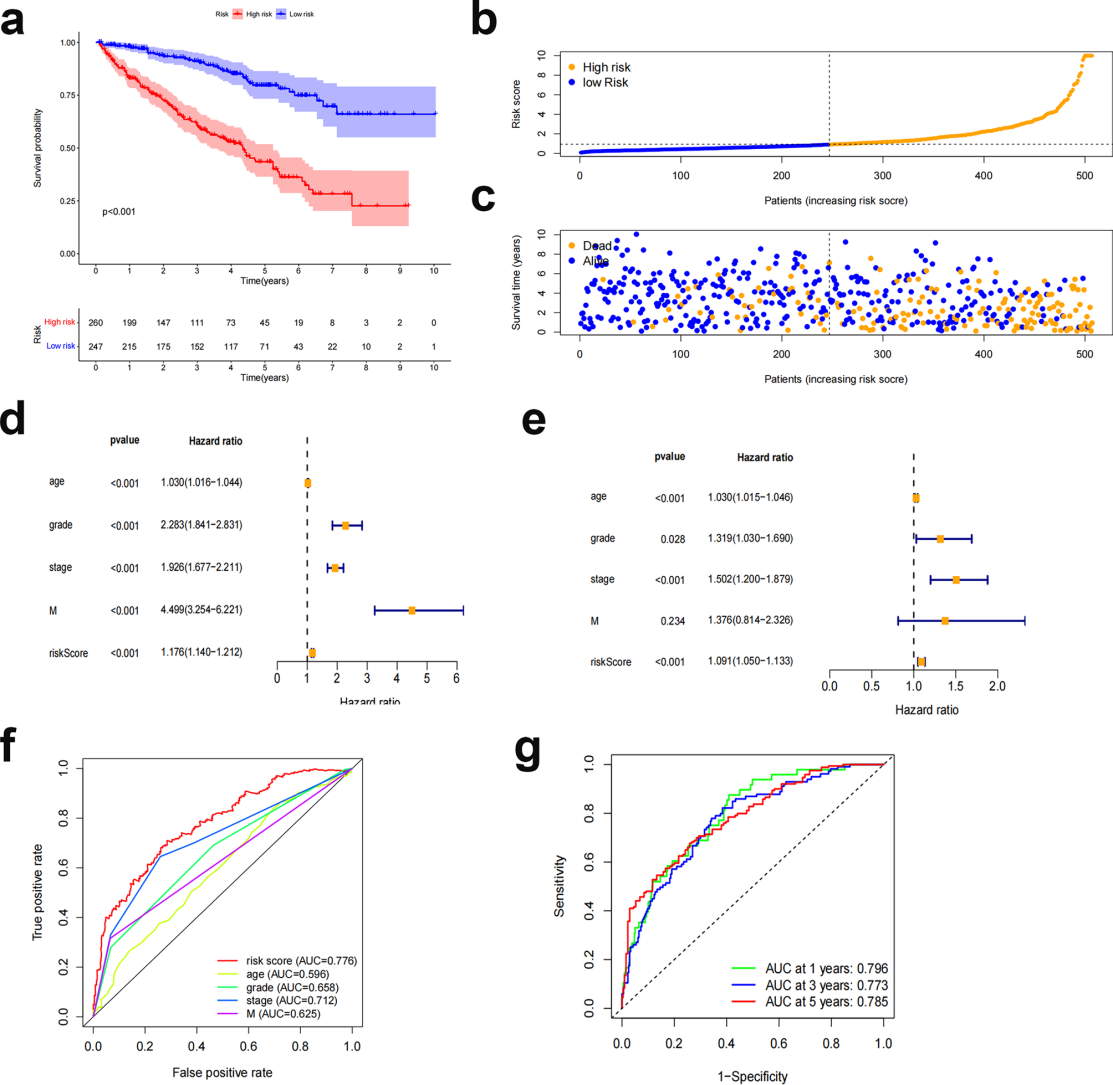
Correlation between prognostic features and prognosis for ccRCC patients. (**a**) Kaplan‒Meier analysis of OS rates for patients with ccRCC in high- and low-risk groups. (**b**) Distribution of risk scores among patients with ccRCC. (**c**) Percentage of patients who died and survived for each risk score. Red denotes the number of fatalities, whereas blue denotes the number of survivors. (**d**) Forest plot of a Cox regression analysis with one variable. (**e**) Multivariate Cox regression analysis forest plot. (**f**) Risk scores versus clinicopathological factors using ROC curves . (**g**) ROC curves and AUCs for 1-year, 3-year, and 5-year survival predictions. AUC, area under the curve; T, tumor; M, metastasis; ROC, receiver operating characteristic; ccRCC, renal clear cell carcinoma.

**Figure 6:**
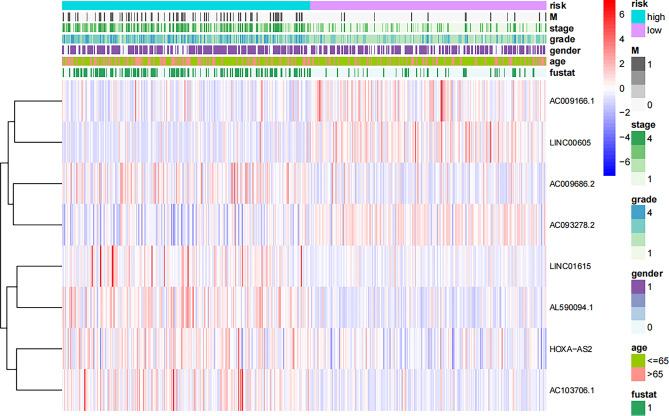
Heatmap (Purple: high expression; brilliant blue: low expression ) for the connections between, clinicopathologic features and the risk groups (R software (version 4.2.1, URL: http://www.r-project.org)). lncRNAs, long noncoding RNAs; T, tumor; M, metastasis.

The risk score of each patient was determined according to the algorithm, and the patients were then separated into high-risk and low-risk groups based on the median value. Kaplan‒Meier analysis of overall survival times revealed that the low-risk group's OS was significantly lower than that of the high-risk group (Fig. 5a, p < 0.001). The difference in risk scores is depicted in Fig. 5b, where it can be seen that as risk scores rise, more deaths occur (Fig. 5c). Age, grading, staging, M-staging, and risk score were significantly associated with OS in ccRCC patients according to one-way Cox regression analysis, and age, grading, staging, and risk score were independent predictors of OS in ccRCC patients according to multiway Cox regression analysis (Fig. 5d), indicating that the risk characteristics were independent risk factors for ccRCC prognosis (Fig. 5e). The predictive value of other clinicopathological variables was lower than the AUC of the risk score (AUC = 0.776) (Fig. 5f). The AUCs for 1-, 3-, and 5-year survival were 0.796, 0.773, and 0.785, respectively, demonstrating good predictive performance (Fig. 5g). We examined the differences in clinicopathological characteristics between the high-risk and low-risk groups to rule out the influence of these factors. However, there was no discernible difference between the groups (Fig. 6).

**Figure 7:**
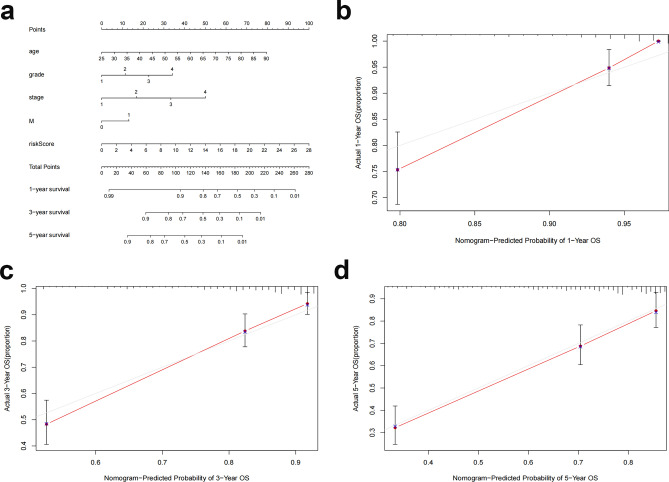
Creation and validation of a nomogram. (**a**) Clinicopathological factors and risk scores combined with a nomogram to predict survival in ccRCC patients at 1, 3, and 5 years. (**b**–**d**) Calibration curves to assess whether expected survival at 1, 3, and 5 years agrees with actual OS rates. M, metastasis; OS, overall survival; ccRCC, clear cell renal cell carcinoma.

To further predict the prognosis of ccRCC patients, we constructed nomogram prediction plots integrating clinicopathological variables and risk scores to predict prognosis at 1, 3, and 5 years (Fig. 7a). Following calibration, the findings revealed that the anticipated survival and actual OS rates were in good agreement (Fig. 7b–d).

### Relationship between predictive characteristics of various clinicopathological indicators and ccRCC patient prognosis

**Figure 8:**
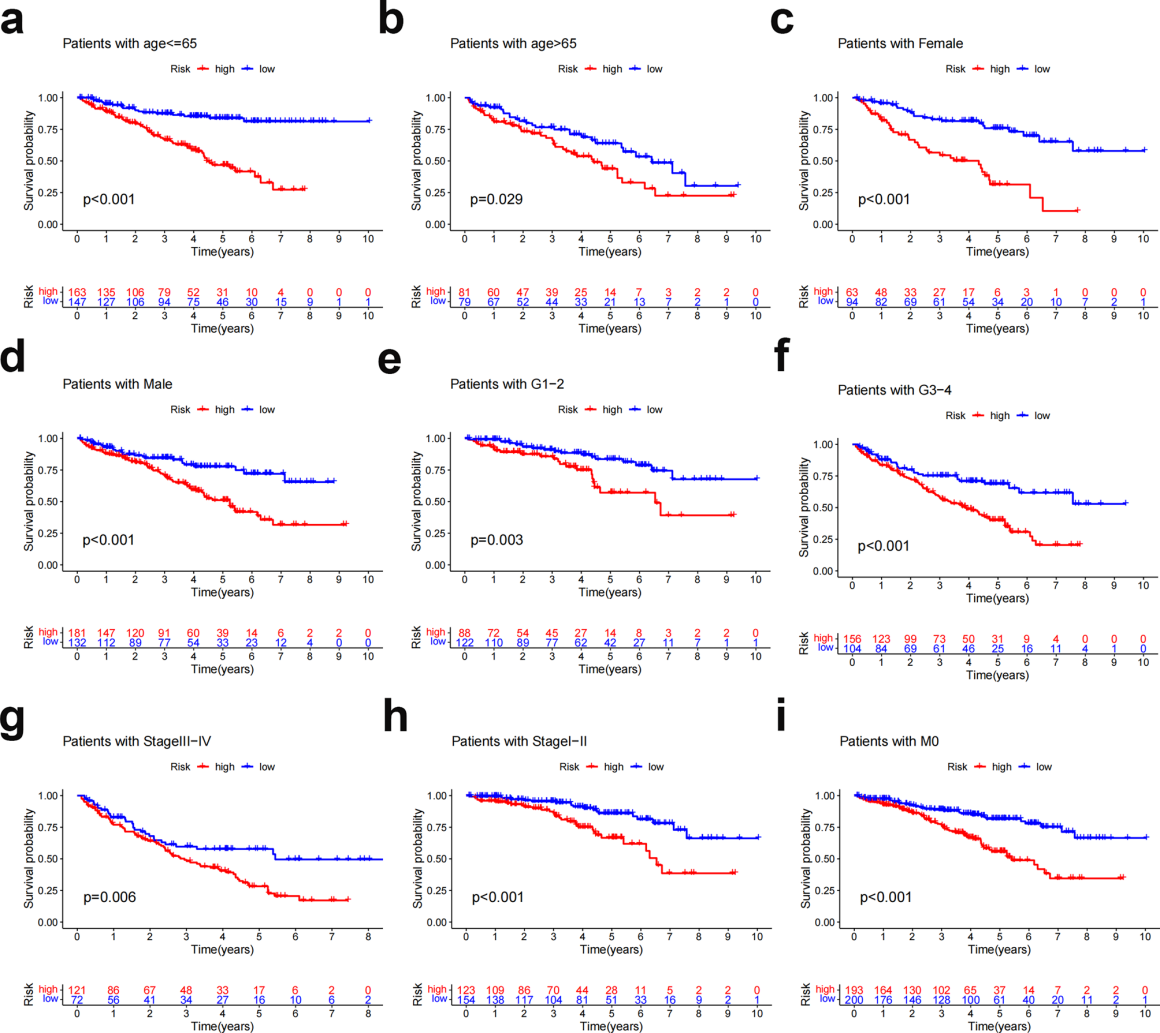
Kaplan‒Meier survival curves for high- and low-risk patients based on clinicopathological factors. (**a**,**b**) Age. (**c**,**d**) Sex. (**e**,**f**) Grading. (**g**,**h**) Staging. (**i**) M staging. M, distant metastases.

To examine the association between prognostic indicators and predictive markers in ccRCC patients under various clinicopathological variable classifications, subgroup analysis of survival was conducted among patients with varying ages, grades, stages and M stages and both sexes. OS was poorer in all high-risk groups than in low-risk groups (Fig. 8), suggesting that predictive characteristics can predict ccRCC prognosis under diverse conditions.

### Internal validation of predictive characteristics

**Figure 9:**
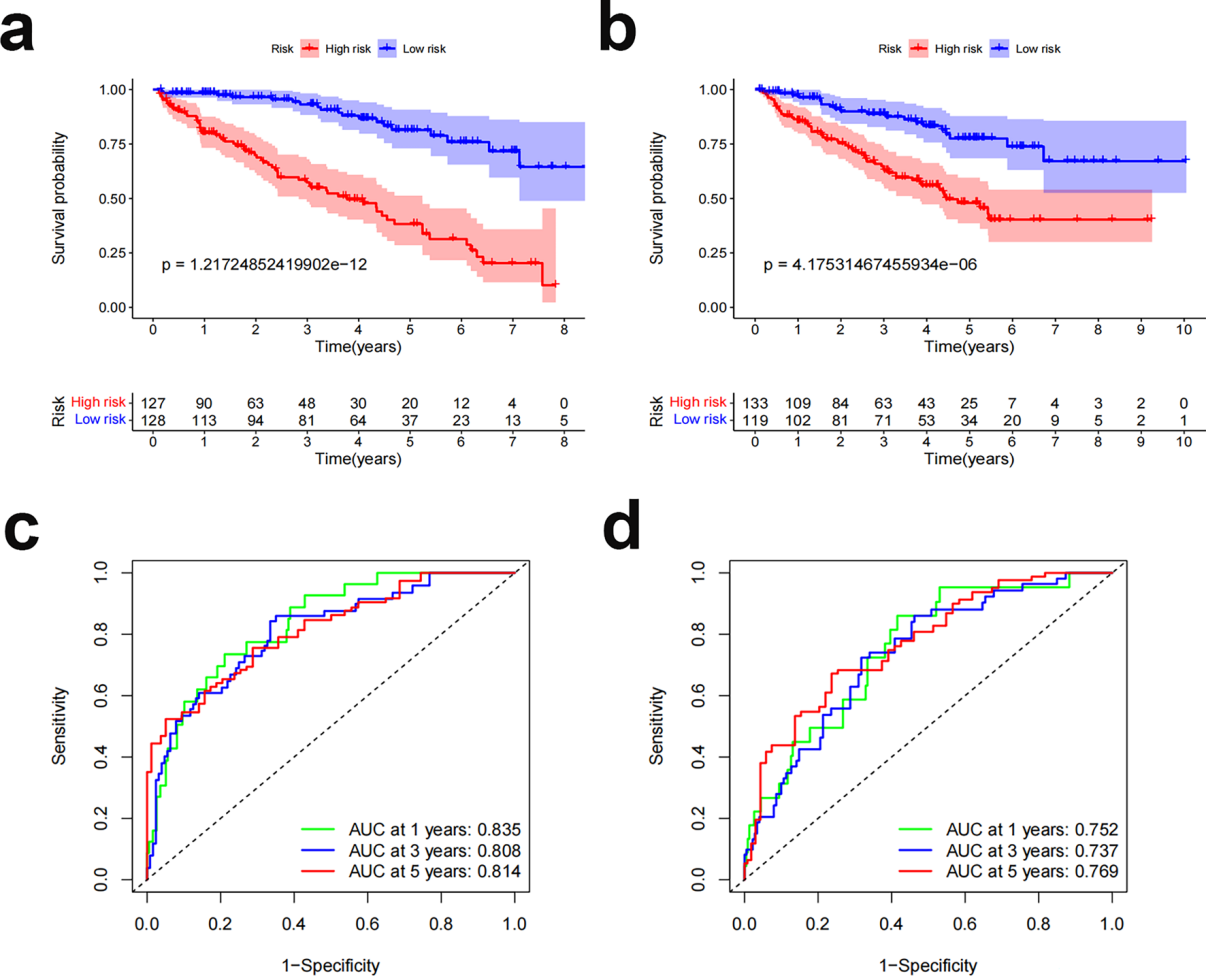
Internal validation of OS prediction signatures based on internal datasets. (**a**) Training group Kaplan‒Meier survival curves. (**b**) Test group Kaplan‒Meier survival curves. (**c**) ROC curves and AUCs for 1-, 3-, and 5-year survival in the training group. (**d**) ROC curves and AUCs for 1-, 3-, and 5-year survival in the test group. ROC, receiver operating characteristic; AUC, area under the curve; OS, overall survival.

We randomly divided ccRCC patients into two groups to verify the applicability of the prognostic prediction signature based on the whole TCGA dataset; the demographic characteristics are displayed in Table 1. The high-risk group of the training cohort had worse survival than the low-risk group, in line with the findings for the overall dataset (Fig. 9a, p = 1.22e−12). In addition, the OS rate was lower in the high-risk group than in the low-risk group in the validation cohort (Fig. 9b, p = 4.17e−06). ROC curves for both groups provided an indicator of the patient's clinical performance. AUCs for the training group's 1-, 3-, and 5-year survival rates were 0.835, 0.808, and 0.814, respectively (Fig. 9c); those for the validation group's 1-, 3-, and 5-year survival rates were 0.752, 0.737, and 0.769, respectively (Fig. 9d).

**Table 1:** Clinical characteristics of the different cohorts of patients.

Variables	Entire TCGA dataset (n = 507 )	Internal validation cohort	
		First cohort (n = 255)	Second cohort (n = 252)
Age (%)			
≤ 65	336 (66.3)	165 (64.7)	171 (67.9)
> 65	171 (33.7)	90 (35.3)	81 (32.1)
Gender (%)			
Female	174 (34.3)	91 (35.7)	83 (32.9)
Male	333 (65.7)	164 (64.3)	169 (67.1)
Grade (%)			
G1 + 2	227 (44.8)	115 (45.1)	112 (44.4)
G3 + 4	272 (53.6)	138 (54.1)	134 (53.2)
GX + unknown	8 (1.6)	2 (0.8)	6 (2.4)
Stage (%)			
I + II	306 (60.3)	151 (59.2)	155 (61.5)
III + IV	198 (39.1)	101 (39.6)	97 (38.5)
TX + unknown	3 (0.6)	3 (1.2)	0 (0)
T (%)			
T1 + 2	324 (63.9)	160 (62.7)	164 (65.1)
T3 + 4	183 (36.1)	95 (37.3)	88 (34.9)
M (%)			
M0	392 (77.3)	202 (79.2)	190 (75.4)
M1	73 (14.4)	37 (14.5)	36 (14.3)
MX + unknown	42 (8.3)	16 (6.3)	26 (10.3)
N (%)			
NO	16 (3.2)	10 (4.0)	6 (2.4)
N1	266 (52.4)	135 (52.9)	131 (52.0)
NX	225 (44.4)	110 (43.1)	115 (45.6)

T, tumor; M, metastasis; N, lymph node.

### Infiltration of immune cells and functional analysis

**Figure 10:**
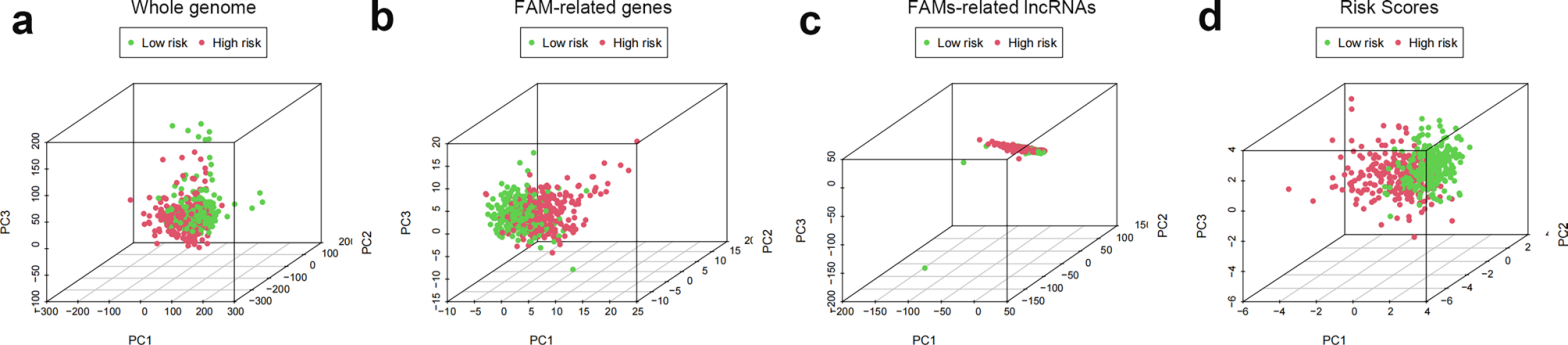
PCA profiles showing patient distribution based on (**a**) genome-wide; (**b**) zinc finger-associated genes; (**c**) zinc finger-associated lncRNAs; and (**d**) risk scores. The separation between red and green dots is stronger in the high- than in the low-risk group.

**Figure 11:**
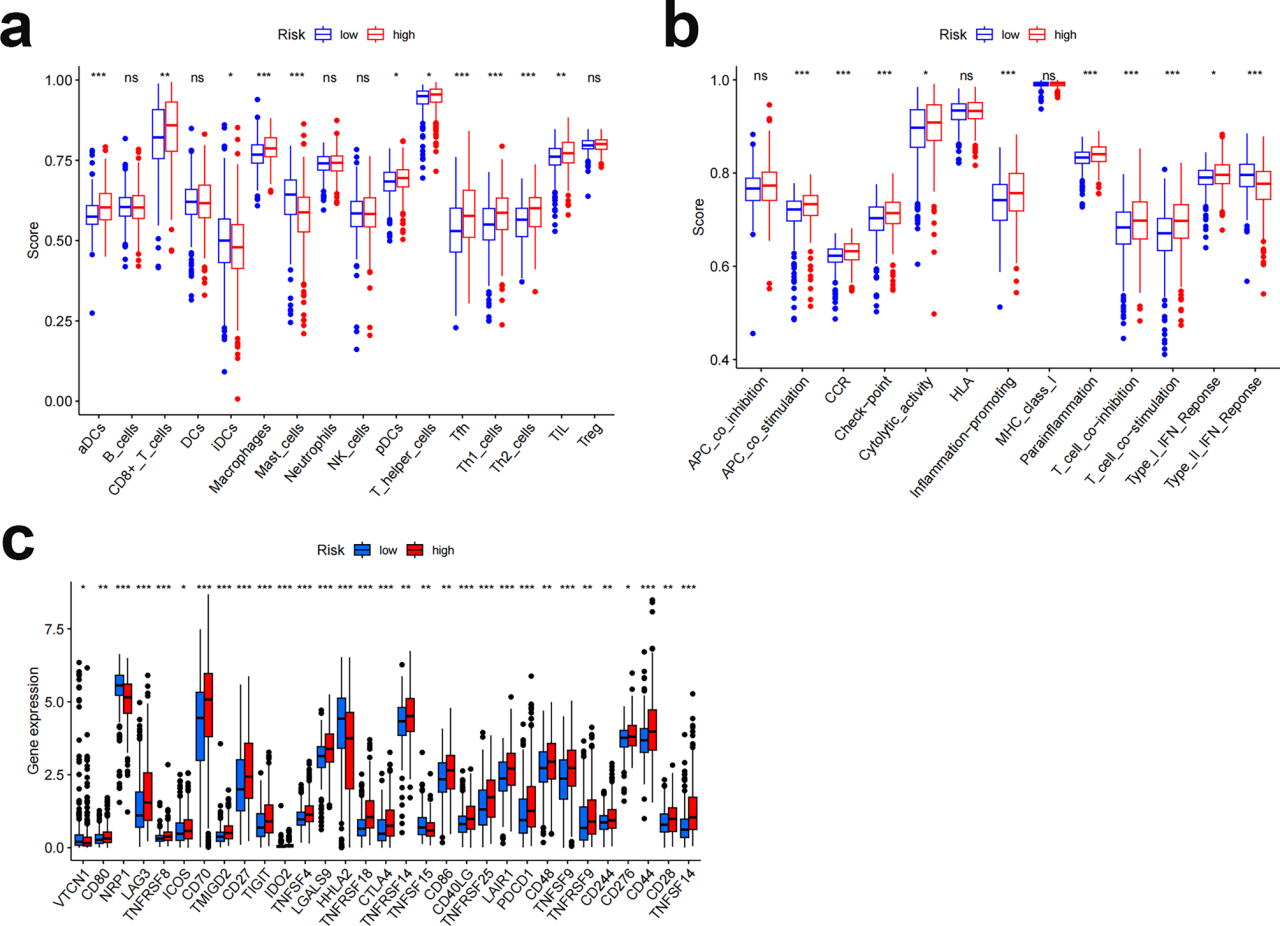
Immuno-infiltration analysis. Results of ssGSEA scoring. (**a**) Sixteen immune cell scores. (**b**) Thirteen immune-related function scores. (**c**) Expression of immune checkpoints in high- and low-risk populations. ssGSEA, single-sample gene set enrichment analysis; aDCs, activated dendritic cells; iDCs, immature dendritic cells; NKs, natural killer cells; pDCs, plasmacytoid dendritic cells; Tfh, T follicle helper cell; Th1, T helper factor type 1; Th2, T helper cell type 2; TIL, tumor-infiltrating lymphocyte; Treg, T-regulatory cell; APC, antigen-presenting cell; CCR, chemokine receptor; HLA, human leukocyte antigen; MHC, major histocompatibility complex; IFN, interferon. *p < 0.05; **p < 0.01; ***p < 0.001; ns, not significant.

To illustrate the spatial distribution of high- and low-risk samples, we visualized the distribution of patients based on the complete genome, FAM-associated gene sets, FAM-associated lncRNAs, and predictive characteristics using principal component analysis (PCA). High- and low-risk patients grouped into separate quadrants based on predictive factors (Fig. 10). As TME composition is an essential indicator of the development of cancer, we evaluated the distribution of several immune cell subtypes in the two risk groups using several genetic markers. The results showed that patients in the high- and low-risk groups had significantly different levels of activated dendritic cells (aDCs), CD8+ T cells, immature dendritic cells (iDCs), mast cells, macrophages, T helper cells, T follicular helper (Tfh) cells, T helper type 1 (Th1) cells, T helper type 2 (Th2) cells, and tumor-infiltrating lymphocytes (TILs) (Fig. 11a). Moreover, the high-risk group had higher immune function scores for chemokine receptors (CCR), checkpoints, lysis activity, inflammation promotion, part inflammation, T-cell coinhibition, T-cell costimulation, and type I IFN response (Fig. 11b), indicating an immunologically active state. Furthermore, immune checkpoint expression differed between the groups (Fig. 11c).

### Predictive characteristics and ccRCC therapy correlation

**Figure 12:**
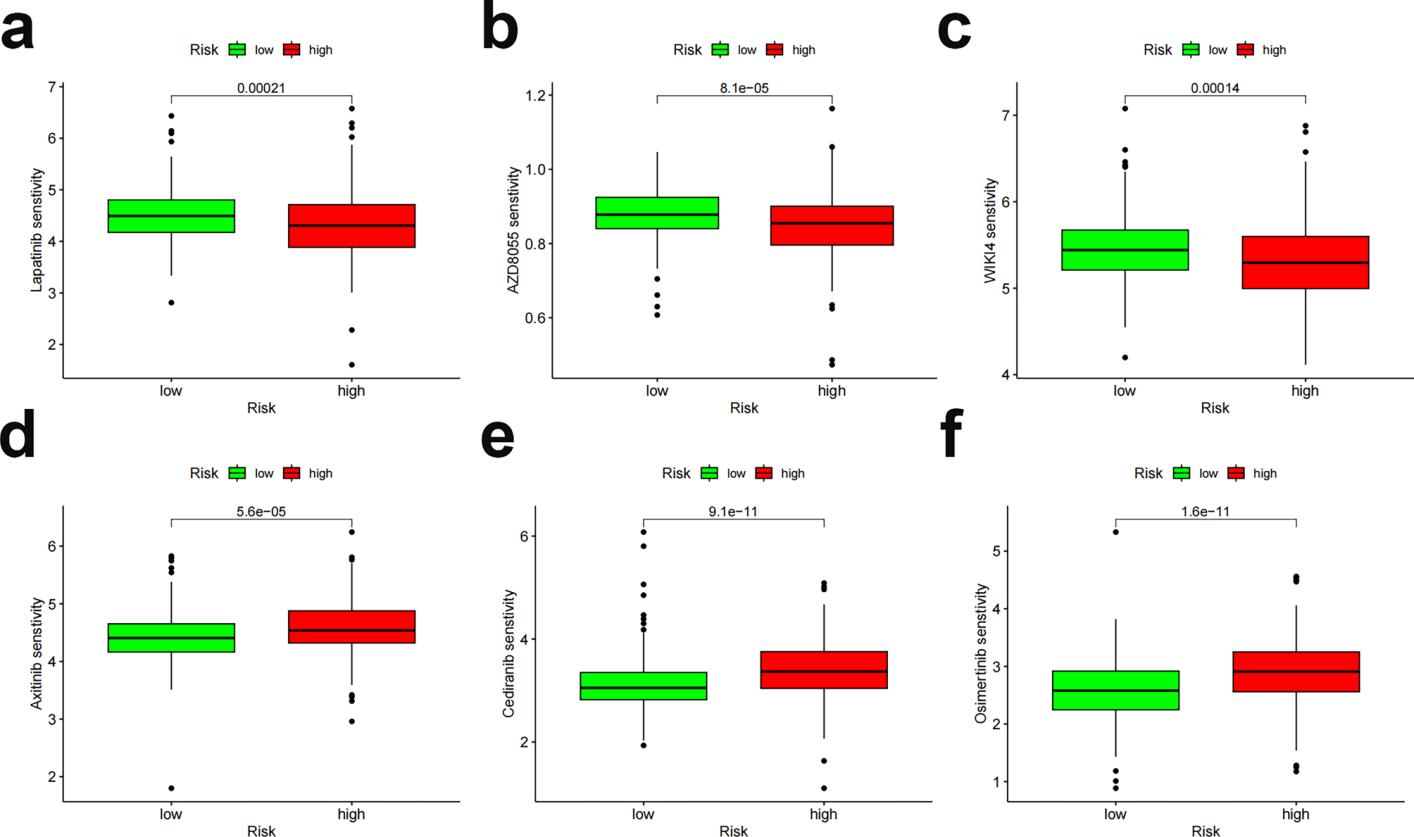
Drug sensitivity analysis. (**a**–**c**) Predicted sensitivity scores for lapatinib, AZD8055, and WIKI4 as chemotherapy candidates for patients with high FAM-related lncRNA scores. (**d**–**f**) Axitinib, cediranib, and osimertinib are candidates for patients with low scores. *p < 0.05; **p < 0.01; ****p < 0.0001.

We utilized the OncoPredict package to predict drug sensitivity scores in the high- and low-risk groups, and sensitivity ratings were favorably associated with chemotherapeutic medication IC50 values. The high-risk population showed strong sensitivity to lapatinib, AZD8055, and WIKI4 (Fig. 12a–c). In the low-risk group, sensitivity to axitinib, cediranib, and osimertinib was high (Fig. 12d–f). Overall, differentiating between high- and low-risk patients can help in developing a personalized treatment plan.

### Correlation between risk scores/FAM genes and clinical variables

**Figure 13:**
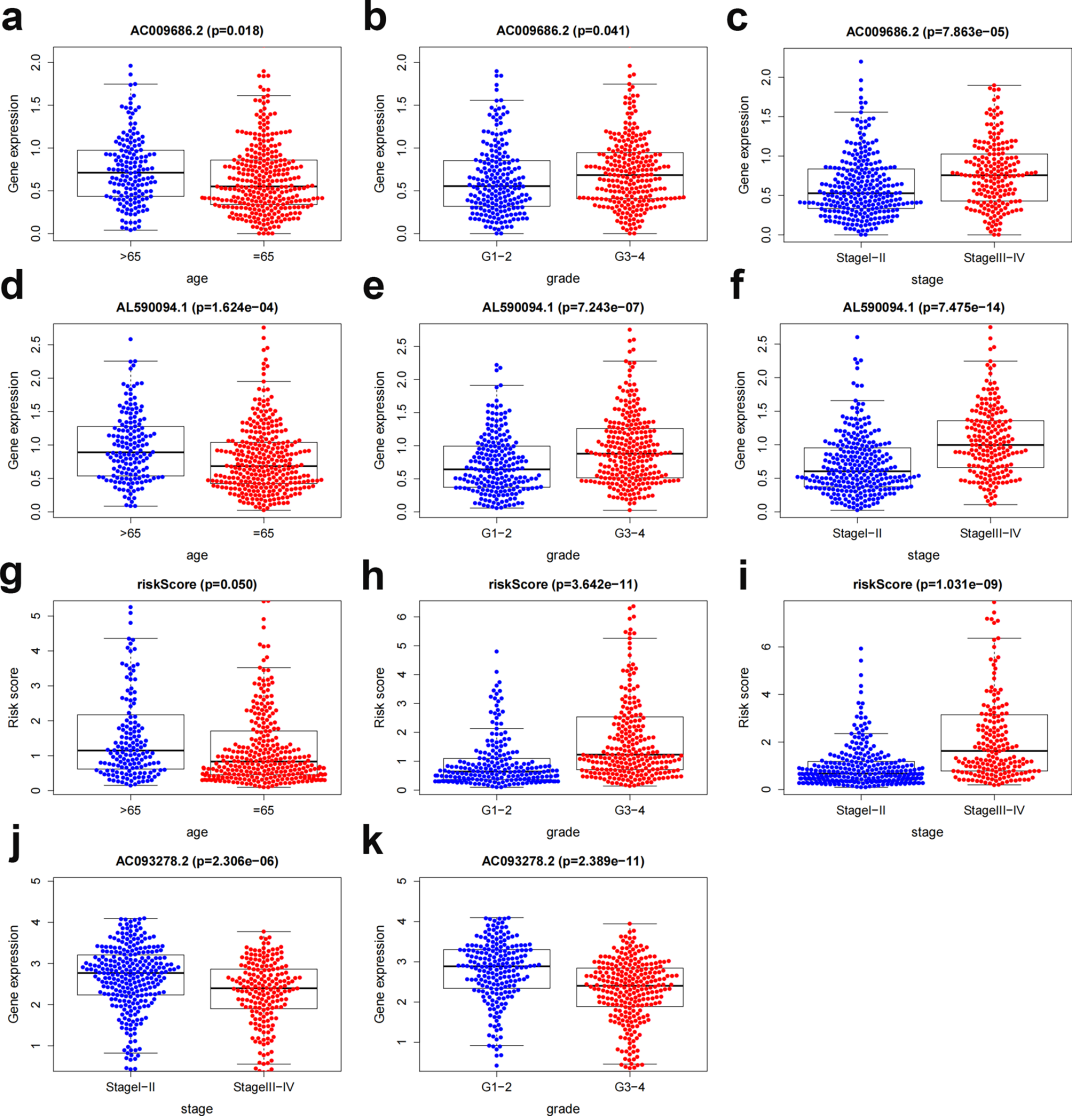
Correlation analysis between model genes and clinical features. (**a**–**c**) Correlation of AC009686.2 expression levels with age, grade, and stage. (**d**–**f**) Correlation of AL590094.1 expression levels with age, grade, and stage. (**g**–**i**) Correlation of risk score with age, grade, and stage. (**j**,**k**) AC093278.2 Correlation of expression level with tumor grade and stage.

We examined the relationship between the above clinical factors and risk ratings of the eight FAM genes based on gene expression and associated clinical data we collected from TCGA. The results demonstrated that AC009686.2, AL590094.1, and risk scores were connected with age, tumor stage, and tumor grade and that AC093278.2 correlated with tumor stage and tumor grade (Fig. 13).

## Discussion

Renal cancer is a heterogeneous disease. According to the latest research, it is thought that the majority of ccRCC cases are caused by a combination of mechanisms, including dysregulation of hypoxia-inducible factor (HIF) signaling, mutations in vital histone and chromatin-modifying enzymes, and metabolic reprogramming of cellular metabolism^27,28^. Despite improvements in targeted therapy and diagnostic methods in recent years, metastasis and invasion in malignancies such as ccRCC can result in a gravely unfavorable prognosis^29^. Sunitinib, a broad-spectrum small-molecule inhibitor of receptor tyrosine kinases (RTKs), is currently the standard treatment for first-line treatment of advanced clear cell renal cell carcinoma (ccRCC). However, the majority of patients develop resistance and experience disease progression^30,31^.

Accumulating data show that angiogenesis, growth, proliferation, and aggressiveness of cancer cells all strongly correlate with metabolic instability^32,33^. According to preliminary data, the physiological behavior of human malignancies is thought to be related to aberrant glycolytic metabolism^34^. In colorectal cancer, abnormal anaerobic metabolic pathways are crucial for development of tumor stem cells (CSCs), which promote rapid tumor development, progression, and medication resistance^35,36^. In addition, cellular energy production, membrane synthesis, and signaling pathways linked to tumor formation are all impacted by FAM^32,37^. Fatty acid anabolism/catabolism dysregulation may promote development of cancerous cells^10^. Previous research has shown that the glutathione redox system prevents the ccRCC iron mortality induced by poor lipid metabolism^38^, and a recent study revealed that FE2F1 activates SREBP1-dependent fatty acid production to increase proliferation and metastasis of ccRCC^39^. Although several researchers have concentrated on the role that FAM plays in different tumors, it is still unclear how this issue applies to ccRCC. It may be possible to gain new knowledge about the biological behavior of these tumors and identify novel and more potent therapeutic approaches by revealing important molecular markers linked to FAM and investigating their role in the development of ccRCC.

In the current work, we thoroughly investigated the function of FAM genes in ccRCC. First, the PI3K-Akt, HIF-1, and other tumor-related signaling pathways were found to be primarily enriched among DEGs; thus, FAM genes with differential expression are linked to tumor growth. Then, we chose the genes in the gray modules that were highly related to tumors for ensuing analysis after using WGCNA to identify four modules. We uncovered eight important FAM genes that may serve as predictive biomarkers in clinical management of ccRCC patients, as based on LASSO and multivariate Cox regression analysis. Through univariate and multivariate Cox regression analyses, a prognostic risk score model that divide patients into high-risk and low-risk categories was produced, with the prognostic risk score as an independent prognostic factor. The model's prediction ability was validated with risk assessment nomogram clinical characteristics (age, sex, and tumor TNM stage). Here, we present a risk model that may be used to identify ccRCC patients with poor prognosis and to manage this disease.

Based on a literature review, these eight genes are somewhat related to cancers, and five genes linked explicitly to ccRCC were found among them according to PubMed. For instance, it has been revealed that the tumor-infiltrating lymphocyte-associated long noncoding RNA LINC01615 is involved in predicting ccRCC^40^. By controlling several lncRNAs, sorafenib plays a role in the response to sorafenib in several cancer cell types^41^. Among three additional genes, AC103706.1 may be able to determine the prognosis of individuals with renal subhyalinosis^42^, and the ccRCC immune microenvironment and prognosis can be predicted by the ferroptosis-related long noncoding RNA AL590094.1^43^. Ac093278.2, an immune-related long noncoding RNA, is used to predict prognosis in ccRCC^44^. A PubMed search for "GENE and ccRCC" revealed that the three genes AC009166.1, LINC00605, and AC009686.2 have not been investigated in ccRCC. Additional fundamental and clinical studies are necessary to confirm the mechanisms underlying the predictive value of the eight genes on which our observations and risk models are based, as well as to develop new therapeutic targets to improve OS in ccRCC patients.

Many researchers have reported the relationship between FAM and the TME. For instance, under hypoxic conditions, Tregs in glioblastoma depend on fatty acid oxidation (FAO) for energy^45^. Significant CD8+ T-cell infiltration has been linked to clinical outcomes and immunological response in ESCA, according to earlier research^46,47^. An elevated CD8+ T-cell to Treg ratio has been linked to poor prognosis in RCC^48^. In samples from individuals with ccRCC in the high-risk category, we noted a rise in infiltrating CD8+ T cells, indicating poor prognosis. Furthermore, poor prognosis is linked to advanced thyroid carcinoma and high tumor-associated macrophage infiltration^49^. The high-risk group also had greater type I IFN response scores and less antitumor immunity in addition to increased tumor immune cell infiltration. IFN production is a major regulator of PD-L1 expression in tumor cells and host cells, and it may enhance the benefit of anti-PD-1 therapy^50^. Therefore, a diminished antitumor immune response may portend worse outcome for those at risk. In general, patients with advanced cancers have a better chance of surviving with checkpoint inhibitor-based immunotherapy^51^.

To our knowledge, this work is the first investigation of prognostic FAM-related lncRNAs in ccRCC. The signature, which is based on eight FAM-related lncRNAs, offers a practical way to predict clinical outcome and offers some guidance for choosing immunotherapeutic and chemotherapeutic treatments. However, there are a number of limitations that should be considered. First, we solely used data from TCGA for internal validation, and external validation requires data from other databases to confirm the applicability of the prediction signature. Second, we used information from public databases as retrospective data. Therefore, additional real-world data in the future are required to confirm therapeutic applicability. In addition, our research lacks validation by in vivo and in vitro experiments. The next step is to confirm how FAM-related lncRNAs function in ccRCC.

## Conclusion

In conclusion, our FAM-associated lncRNA signature can independently predict prognosis of patients with ccRCC. Furthermore, it offers a potentially fruitful route for selecting antitumor immunotherapy and chemotherapeutic drugs.

## Methods

### Data acquisition and processing

Through TCGA (https://portal.gdc.cancer.gov/), we accessed RNA-seq data with FPKM normalization for renal clear cell carcinoma (TCGA- KIRC) together with related clinical and prognostic data. lncRNA expression and survival time data were gathered for 611 patients. Patients with less than 30 days of follow-up were excluded; 507 patients met the inclusion requirements. The patients were randomly assigned in a 1:1 ratio to a training group (n = 255) and test group (n = 252). GeneCards (https://www.genecards.org) provided download access to 9368 genes involved in FAM.

### Screening for DEGs

First, FAM genes in GeneCards with correlation values below 7 were disregarded. Then, DEGs were obtained by comparing normal and ccRCC tissues in TCGA and the filter criteria using a false discovery rate (FDR) of < 0.05 and |log2-fold change (FC)| ≥ 1^52^. Nine hundred-seven differentially expressed FAM-related genes were subsequently identified for further investigation.

### Functional enrichment analysis

Using the "ggplot2" package, we carried out analyses using the Gene Ontology (GO) and Kyoto Encyclopedia of Genes and Genomes (KEGG) databases. We found mRNAs that had strong correlations with the 8 FAM-related lncRNAs mentioned above while assessing the biological function of these lncRNAs in ccRCC. Five coexpression networks of lncRNAs and mRNAs were created and visualized with Sankey plots. Correlation coefficient criteria were set at > 0.4 or -0.4, and P values < 0.001 were deemed statistically significant.

### WGCNA

According to an earlier description^53^, ccRCC-related modules were identified using the WGCNA (version 1.61) package. To summarize, soft thresholds were calculated using a scale-free topology criterion. The minimal module size was set to 50 genes, and the ideal soft threshold was selected. Dynamic tree cuts with the MEDissThres option set to 0.25 were used to identify modules.

### Prediction characteristics of FAM-related lncRNAs

Using "limma" software, we determined the association between FAM-related genes and lncRNAs. Applying the filters |R2| > 0.4 and p < 0.001, a total of 1258 FAM-related lncRNAs were retrieved along with their associated expression data. We initially performed one-way Cox regression analysis to obtain FAM-related lncRNAs linked to prognosis in ccRCC patients. Then, we used LASSO Cox regression analysis to obtain FAM-related lncRNAs to create predictive features. Risk score = beta i * expression i, where beta i is the coefficient and i is the lncRNA, was applied to define the prognostic model. In both datasets, risk scores were computed for each sample. The samples were split into high-risk and low-risk groups based on the median risk score, which served as the cutoff point. The survivor and survminer R packages were employed to conduct Kaplan‒Meier survival analysis to compare the survival rates of the two groups. Clustering of risk characteristics was visualized using principal component analysis (PCA) utilizing "Limma" and "scatterplot3d"^54,55^. Additionally, univariate and multivariate Cox regression analyses were carried out to evaluate the association between clinical variables and risk scores. The timeROC package in R was utilized to validate the accuracy and capacity of prediction^56^. All approaches were included in an internal validation cohort to confirm the applicability of the predictive signature.

### Creating column line diagrams

We created nomogram survival charts that predicted 1-, 2-, 3-, and 5-year survival in patients with ccRCC by combining risk scores with age, sex, stage, and stage M clinicopathological features. Calibration curves were then applied to determine whether the predicted survival is compatible with actual survival.

### Drug sensitivity analysis with predictive signatures

We assessed the contribution of predictive features in predicting response to ccRCC treatment using the Genomics of Drug Sensitivity in Cancer (GDSC) database. This public dataset collects information on therapeutic sensitivity and molecular indicators of drug response in cancer cells^57^. OncoPredict was used to download GDSC2 gene expression profiles and related drug response information^58^. We used sensitivity ratings to estimate the half-maximal inhibitory concentration (IC50) of each medicine in ccRCC patients.

### Quantification of immune infiltration levels

The "gsa" program was utilized to compare immune cells and pathways between the two groups utilizing ssGSEA. Using the "GSVA" program, single-sample gene set enrichment analysis (ssGSEA) was employed to determine the infiltration score of 16 immune cells and activation of 13 immune-related pathways^59^. Finally, we investigated the correlation between risk scores and immunological checkpoints by contrasting gene expression levels between high-risk and low-risk groups.

### Statistical analysis

R software (version 4.2.1, http://www.r-project.org) was used to conduct all statistical analyses. Expression levels of DEGs related to FAM in healthy and malignant tissues were compared using the Wilcoxon test. FAM-related lncRNAs were discovered to be an independent prognostic risk factor for ccRCC based on univariate and multivariate Cox models. The area under the curve (AUC) of the receiver operating characteristic (ROC) curve was used to determine how accurately the FAM-related lncRNA model predicts ccRCC. All statistical tests were two-sided, with p < 0.05 regarded as statistically significant. Statistical analyses were carried out using the R environment and the Bioconductor package (version 3.5.5).

## Supplementary Information

Supplementary Legends.

Supplementary Information 2.

Supplementary Information 3.

Supplementary Information 4.

Supplementary Information 5.

## Data Availability

The corresponding author will gladly provide access to the datasets used and analyzed during the current work upon reasonable request.
