# Supplementary material for: Identification and validation of fatty acid metabolism-related lncRNA signatures as a novel prognostic model for clear cell renal cell carcinoma
Source: Sci Rep. 2023 Apr 29;13:7043. doi: 10.1038/s41598-023-34027-9 (PMC10148808; doi:10.1038/s41598-023-34027-9)
Supplement: Supplementary file 1 — Supplementary Legends. [file 41598_2023_34027_MOESM1_ESM.docx]

**Additional information**

Supplementary File 1 | FAM-related differentially expressed genes

Supplementary File 2 | FAM-related differentially expressed genes in the gray module

Supplementary File 3 | Differentially expressed FAM-associated lncRNAs

Supplementary File 4 | Eight FAM-associated lncRNAs in the model
